# Lack of durable protection against cotton smoke-induced acute lung injury in sheep by nebulized single chain urokinase plasminogen activator or tissue plasminogen activator

**DOI:** 10.1186/s40169-018-0196-3

**Published:** 2018-06-18

**Authors:** Satoshi Fukuda, Perenlei Enkhbaatar, Christina Nelson, Robert A. Cox, Marla R. Wolfson, Thomas H. Shaffer, Robert O. Williams, Soraya Hengsawas Surasarang, Sahakijpijarn Sawittree, Galina Florova, Andrey A. Komissarov, Kathleen Koenig, Krishna Sarva, Harrison T. Ndetan, Karan P. Singh, Steven Idell

**Affiliations:** 1 Translational Intensive Care Unit, Department of Anesthesiology, The University of Texas Medical Branch, Galveston, TX USA; 2 Department of Physiology, Lewis Katz School of Medicine, Temple University, Philadelphia, PA USA; 3 Department of Thoracic Medicine and Surgery, Lewis Katz School of Medicine, Temple University, Philadelphia, PA USA; 4 Center for Inflammation, Translational and Clinical Lung Research, Lewis Katz School of Medicine, Temple University, Philadelphia, PA USA; 5 CENTRe: Collaborative for Environmental and Neonatal Therapeutics Research, Lewis Katz School of Medicine, Temple University, Philadelphia, PA USA; 6 Temple Lung Center, Lewis Katz School of Medicine, Temple University, Philadelphia, PA USA; 7 Center for Pediatric Lung Research, Alfred I. DuPont Hospital for Children, Wilmington, DE USA; 8 Molecular Pharmaceutics and Drug Delivery, The University of Texas at Austin, Austin, TX USA; 9 The Department of Cellular and Molecular Biology and the Texas Lung Institute, The University of Texas Health Science Center at Tyler, 11927 US HWY 271, Tyler, TX 75708 USA; 10 The Department of Epidemiology and Biostatistics, The University of Texas Health Science Center at Tyler, 11927 US HWY 271, Tyler, TX 75708 USA

**Keywords:** Plasminogen activators, Fibrinolysins, Plasminogen activator inhibitor-1, Inhalational acute lung injury

## Abstract

**Background:**

Airway fibrin casts are clinically important complications of severe inhalational smoke-induced acute lung injury (ISIALI) for which reliable evidence-based therapy is lacking. Nebulized anticoagulants or a tissue plasminogen activator; tPA, has been advocated, but airway bleeding is a known and lethal potential complication. We posited that nebulized delivery of single chain urokinase plasminogen activator, scuPA, is well-tolerated and improves physiologic outcomes in ISIALI. To test this hypothesis, we nebulized scuPA or tPA and delivered these agents every 4 h to sheep with cotton smoke induced ISIALI that were ventilated by either adaptive pressure ventilation/controlled mandatory ventilation (APVcmv; Group 1, n = 14) or synchronized controlled mandatory ventilation (SCMV)/limited suctioning; Group 2, n = 32). Physiologic readouts of acute lung injury included arterial blood gas analyses, PaO_2_/FiO_2_ ratios, peak and plateau airway pressures, lung resistance and static lung compliance. Lung injury was further assessed by histologic scoring. Biochemical analyses included determination of antigenic and enzymographic uPA and tPA levels, plasminogen activator and plasminogen activator inhibitor-1 activities and d-dimer in bronchoalveolar lavage (BAL). Plasma levels of uPA, tPA antigens, d-dimers and α-macroglobulin-uPA complex levels were also assessed.

**Results:**

In Group 1, tPA at the 2 mg dose was ineffective, but at 4 mg tPA or scuPA, the PaO_2_/FiO_2_ ratios, peak/plateau pressures improved during evolving injury (p < 0.01) without significant differences at 48 h. To improve delivery of the interventions, the experiments were repeated in Group 2 with limited suctioning/SCMV, which generally increased PAs in (BAL). In Group 2, tPA was ineffective, but scuPA (4 or 8 mg) improved physiologic outcomes (p < 0.01) and plateau pressures remained lower at 48 h. Airway bleeding occurred at 8 mg tPA. BAL plasminogen activator (PA) levels positively correlated with physiologic outcomes at 48 h.

**Conclusions:**

Physiologic outcomes improved in sheep in which better delivery of the PAs occurred. The benefits of nebulized scuPA were achieved without airway bleeding associated with tPA, but were transient and largely abrogated at 48 h, in part attributable to the progression and severity of ISIALI.

**Electronic supplementary material:**

The online version of this article (10.1186/s40169-018-0196-3) contains supplementary material, which is available to authorized users.

## Background

Smoke exposure can induce fibrinous airway cast formation and inhalational smoke-induced acute lung injury (ISIALI), a feared and potentially life-threatening complication for which optimal therapy is currently unclear [1]. The precise incidence of this problem is also unclear but there are about a million Americans who are annually exposed to smoke from fires, of whom a relatively small proportion develop ISIALI. This complication contributes to an estimated 3000 deaths annually [2]. In ISIALI, airway cast formation is initiated by damage to the airway epithelial lining, where robust airway coagulation occurs contingent upon ongoing leakage of plasma substrates into the large airways. The deposition and persistence of these casts in the airways suggests that endogenous fibrinolytic activity is locally impaired. Supportive care is now the mainstay of therapy and the mortality of ISIALI remains high [3]. Anticoagulant strategies including administration of nebulized heparins, other anticoagulants or use of fibrinolysins; plasminogen activators (PAs) or plasmin, have been applied pre-clinically and clinically, but it is unclear as to which option or class of agents is best. The paucity of robust trials has triggered calls for well-designed additional prospective studies to advance the field [4]. Overall, these considerations offer a strong premise for the testing of new interventional approaches for ISIALI and for this study.

Nebulized plasminogen activators conceptually offer great promise for the treatment of airway coagulation. Nebulized recombinant tissue plasminogen activator (tPA; activase/alteplase, Genentech, San Francisco, CA) has previously been reported to improve ISIALI by our group [5], providing proof of concept that this approach could be beneficial. Airway delivery of tPA has also been shown to improve outcomes of mustard gas-induced lung injury [6]. On the other hand, repeated airway administration of escalating doses of tPA have been shown to induce airway bleeding [7], which by extrapolation represents a potentially serious challenge for airway administration of tPA in human subjects. The use of anticoagulants carries similar risks but could mitigate the formation of new casts or facilitate their dissolution by endogenous fibrinolysis over relatively extended periods of time. By their primary mechanism of action, anticoagulants do not directly clear established casts as do fibrinolysins; plasminogen activators, which degrade fibrin deposits by generating increments of plasmin.

We found that a proenzyme, single chain urokinase plasminogen activator, scuPA, effectively clears pleural adhesions and removes organizing fibrinous material from the pleural space [8–10]. In both pleural and airway fluids, scuPA was found to form bioactive complexes with α-macroglobulins (αM), which are resistant to inhibition by plasminogen activator inhibitor-1 (PAI-1) and durably release low levels of active plasminogen activator [11, 12]. Over a 20 years preclinical experience [13], now extended by good laboratory practices (GLP) toxicology done with support from the National Heart Lung and Blood Institute SMARTT Program (Contract HHSN268201100014C), we found that intrapleural use of relatively large doses of intrapleural scuPA were well-tolerated preclinically and did not cause systemic fibrinogenolysis or serious bleeding complications. Based upon these findings, we postulated that scuPA could be advantageous for nebulized delivery to the airway, that it could clear airway casts effectively, improve outcomes and that it would mitigate the risk for local bleeding complications. We used our established model of ISIALI in sheep to test this postulate and compare efficacy and safety outcomes versus those associated with the use of nebulized tPA; activase/alteplase, a commercially available plasminogen activator, or vehicle.

## Methods

### The sheep model of ISIALI

All studies involving animals were approved by the Institutional Animal Care and Use Committees of The University of Texas Medical Branch at Galveston. The same studies and exchange of biological samples were also approved by the Institutional Animal Care and Use Committee of The University of Texas Health Science Center at Tyler. The ovine model of cotton smoke-induced ISIALI in awake animals was used in these studies, as we previously reported [5]. In the model, a total of 48 puffs of cotton bark smoke cooled to < 40 °C in 4 sets of 12 breaths was delivered via the tracheostomy tube by a modified bee smoker to induce ISIALI. A total of 46 sheep with ISIALI were utilized in this study, including those that were ventilated by either adaptive pressure ventilation/controlled mandatory ventilation (APVcmv; Group 1, n = 14) or synchronized controlled mandatory ventilation, (SCMV; Group 2, n = 32) using a Hamilton G5 ventilator (Hamilton Medical, Inc., Reno NV). Interventional agents were nebulized using an Aeroneb^®^ Pro vibrating mesh nebulizer (Aerogen, Mountain View, CA). In addition, another 3 naive (No any interventions i.e., no surgical procedure, no injury, no tracheostomy and ventilation) animals and 2 sham animals ventilated with the APVcmv for 48 h were used as non-ISIALI controls, so that a total of 51 sheep were used in this study. The ventilator parameters included the tidal volume = 12 mL/kg; frequency = 20 breaths/min; Positive end expiratory pressure; peep = 5 cmH_2_O; time of inspiration to total respiratory cycle time; i/e = 1:2; heated/humidified inspired gas to 33 °C, with fractional concentration of oxygen; FiO_2_ = 1, with all parameters constant to 3 h post injury, then titrated to maintain a PaO_2_ ≥ 100 mmHg with frequency adjusted to maintain the PaCO_2_ close to 30–40 mmHg.

**Fig. 1:**
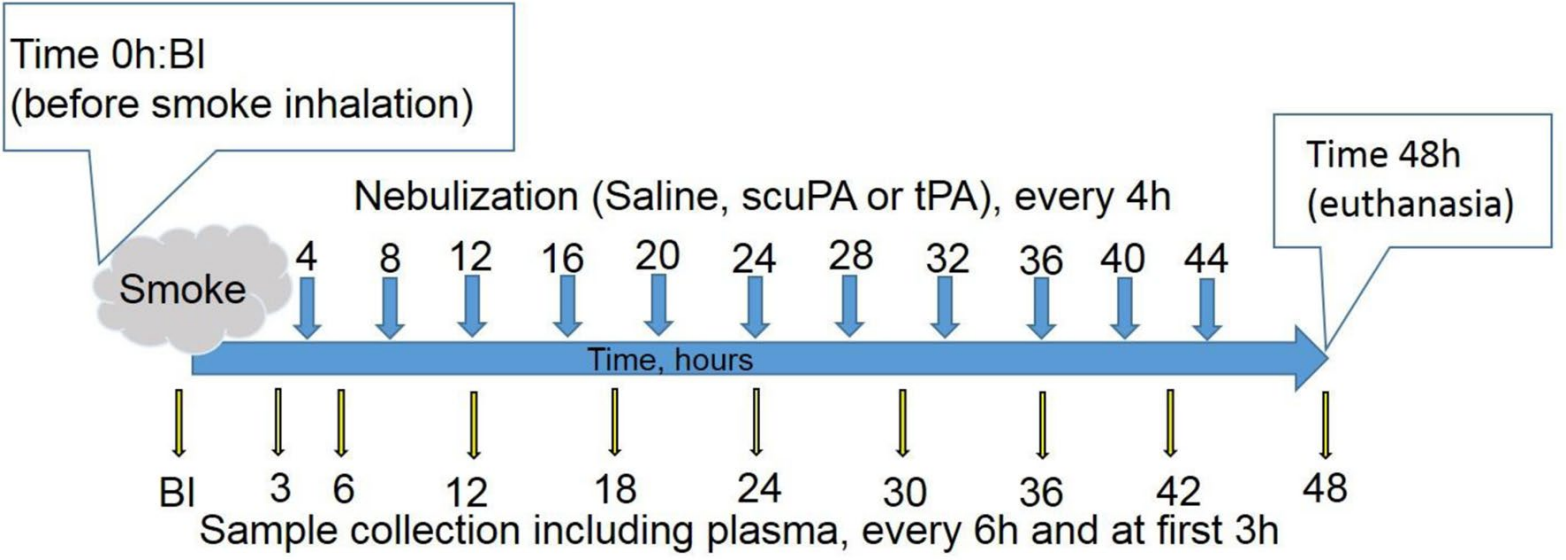
Schematic illustration of the treatment schedule and sample collection protocol. The long blue horizontal arrow defines the 48 h treatment period. The short blue arrows indicate treatment intervals after initiation of ISIALI, which was achieved by the inhalation of 48 successive puffs of cooled cotton smoke, indicated by the smoke cloud depiction. The longer yellow arrows with black borders indicate plasma collection times. *BI* before inhalation

Arterial blood samples were obtained at 3 and 4 h after injury and thereafter every 4 h in Group 1, and 3 h and 6 h after injury and thereafter every 6 h in Group 2 for measurement of arterial blood gas analyses (blood gas analyzer RAPIDPoint 500; Siemens Healthcare, Erlangen, Germany). The PaO_2_/FiO_2_ ratio was measured to assess pulmonary gas exchange. Peak and plateau airway pressures, lung resistance and static lung compliance were measured at these same intervals throughout the 48 h course of the experiments. All physiologic assessments from each of the animals in each of the treatment groups were included. Animals were euthanized if the animals reached euthanasia criteria i.e., PaO_2_/FiO_2_ < 50, PaCO_2_ > 90 mmHg or mean arterial pressure < 50 mmHg sustained for 1 h. A schematic illustration of the sample collection format is depicted in Fig. 1, which illustrates that blood samples from sheep in Group 2 were collected at 3, 6, 12, 18, 24, 30, 36, 42 and 48 h after initiation of ISIALI. Blood was not collected from animals in Group 1. Nebulized interventional agents were delivered at 4, 8, 12, 16, 20, 24, 28, 32, 36, 40, and 44 h after ISIALI was induced, after which the sheep were sacrificed at 48 h.

### Lung preparation, histologic and acute lung injury severity scores

Lung preparation, histologic assessment and scoring of airway obstruction and bleeding were done as we previously reported [5]. Briefly, the right lung was removed, and a 1-cm-thick section was taken from the middle of the lower lobe, injected with and then immersed in 10% formalin. Samples (n = 4) were taken at predetermined sites for histological examination. Fixed samples were embedded in paraffin, sectioned at 4 μm, and stained with hematoxylin and eosin. A pathologist without knowledge of the group assignments evaluated the lung histology. The pathologist then evaluated all bronchi, bronchioles, and respiratory bronchioles in sections of the four tissue samples, and for each, estimated the percentage of the surface area of the lumen obstructed by cast material (0–100%), representing the obstruction score. Other scoring of histopathologic indicators of lung injury were done as we previously reported [5].

### Preparations of tPA and scuPA

Activase [lyophilized alteplase or recombinant single chain (sc); referred to as tPA] was provided as a generous gift from Genentech through the Genentech Research Contracts and Reagents Program to SI. The preparation of scuPA used in all experiments was non-GMP (good manufacturing practices) bulk drug substance that was manufactured under NHLBI SMARTT Contract # HHSN268201100014C. This preparation of scuPA was initially assessed by Advanced Bioscience Laboratories (ABL, Inc). The specific enzymatic activity of the scuPA preparation was about 150,000 IU/mg, and initial purity was assessed by reverse phase high pressure liquid chromatography to be 99.5% scuPA. Bacterial endotoxin levels were low but detectable at 0.23 EU/mg. The aliquoted samples of scuPA were stored in sodium acetate buffer pH 4.5, frozen at − 80 °C and activity and purity of the stored scuPA were validated to be preserved by determination of the specific activity of the material every 6 months.

To assess stability of the fibrinolytic agents during nebulization, samples of 4 mg of single chain tissue plasminogen activator; sctPA or scuPA in 8 mL of PBS were nebulized using an Aeroneb^®^ Pro (Aerogen, Mountain View, CA) and captured for the further stability and activity measurements as described previously [14]. The specific enzymatic activity of the scuPA and sctPA preparations after nebulization were each 95 ± 8% of the measured stock preparation activity. Additionally, the quality of scuPA and tPA preparations used for in vivo experiments was confirmed independently by measuring the fraction of the active enzyme based on its ability to form an inhibitory complex with PAI-1 as described previously [14]. The molar fraction of active enzymes in all preparations was greater than 0.90.

### Bronchoalveolar lavage (BAL) protocol and plasma collection

BAL was accomplished by wedging a Foley urinary catheter within a left lower lobe segment and introducing 50 mL of PBS via the catheter. The harvested return ranged from 5 to 20 mL with gentle suctioning. Cells were removed via centrifugation at 2500*g* at 4 °C for 10 min and cell-free lavage was immediately frozen and stored at − 80 °C. Citrated plasma was collected from animals in Group 2 only as previously reported [8].

### uPA, tPA activity, antigen, fibrinolytic activity, plasminogen activation assays and fibrin enzymography

Measurements of the uPA and tPA activity and antigen in BAL were done using ELISAs (Molecular Innovations, Novi, MI) per the manufacturer’s protocol. Fibrinolytic activity in BAL was analyzed as we previously reported [10]. BAL fluids (0.1 mL) were added over a FITC-fibrin film, which was pre-formed in the 96-well plate, and supplemented with 100 nM of human Glu-plasminogen. Fibrinolytic activity was monitored by fluorescence emission at 510 nm (excitation 490 nm) with time. Plasminogen activation assays were performed as previously described [10]. Fibrin enzymography was done as we previously reported [9].

### PAI-1 activity assays

Levels of active PAI-1 in the BAL samples were determined using an active rat PAI-1 ELISA (Molecular Innovations, Novi, MI) following the manufacturer’s protocol and by titrating samples of BAL fluid with uPA of a known concentration, as previously described [15, 16].

### Effects of nebulization, ventilator circuit, oxygen concentration and humidification on the activity of nebulized enzymes

A ventilator simulation circuit, described in detail and illustrated in Additional file 1: Figure S1, enabled us to assess losses/inactivation of the plasminogen activators.

### Measurements of αM complexes in BAL

αM complexes with either scuPA/uPA or tPA were assessed as we previously reported [14]. Briefly, PAI-1 resistant uPA amidolytic activity, which represents intrapleural uPA in complexes with αMs, was measured after samples of BAL fluids were supplemented with an excess (100–200 nM) of exogenous recombinant human PAI-1 to inhibit free enzyme.

### Statistical analysis

The data was analyzed using SigmaPlot version 12.3 for Windows (Systat Software Inc., San Jose CA) and Graphpad Prism version 6 (Graphpad Software, La Jolla, CA). For each ventilation group and nebulized plasminogen activator dosing modality (at 2, 4 and 8 mg), changes in the physiologic parameters (PaO_2_, PaO_2_/FiO_2_ ratio, peak and plateau airway pressures, resistance and static compliance) between treatment groups (controls, tPA and scuPA) over the 48 h study duration where compared using the Friedman’s 2-way ANOVA by rank, post hoc, where there is statistical significance, by pairwise Wilcoxon signed-rank tests. The impact of changes in the ventilation strategy (Group 1/Group 2) and an increase in dose (from 4 to 8 mg irrespective of treatment type) on the biochemical parameters (BAL and plasma uPA or tPA activity, antigen, active PAI-1) after 48 h of treatment were explored using the Kruskal–Wallis one-way ANOVA on ranks with post hoc using the Dunn’s test. Subgroup correlation analyses were also performed between physiologic and biochemical parameters at specific dosing levels. Statistical significance for each test was assessed at the 5% level of significance.

### Availability of data and datasets

The dataset(s) supporting the conclusions of this article are included within the article and its Additional file 1.

## Results

### Group 1: pathophysiologic responses to ISIALI and to nebulized plasminogen activators treatment

Of the 14 sheep committed to APVcmv mechanical ventilation during the progression of ISIALI (Group 1), the first 2 animals were treated with nebulized 2 mg tPA delivered q4h. Two animals were euthanized because of respiratory and/or hemodynamic compromise at 29 h and 45 h, respectively. While we previously reported that this dose of nebulized tPA delivered every 4 h mitigated physiologic impairment in a similar model of inhalation/burn exposure [5], there was no improvement in the PaO_2_/FiO_2_ ratios, peak or plateau airway pressures, static compliance or airway resistance versus vehicle treated controls (n = 5) throughout the 48 h course of injury. The PaO_2_/FiO_2_ ratios of control saline-treated mechanically ventilated animals with evolving ISIALI generally declined more rapidly than controls included in the 2004 report [5], suggesting that the severity of injury could have contributed to the lack of salutary responses. Because we did not have enough material to provide continuous nebulization, we tested increased; 4 mg, doses of the PAs in the model and compared the effects of treatment with nebulized tPA (n = 4) versus scuPA (n = 3) using the same administration schedule (Fig. 1). One animal in the control group deteriorated and required euthanasia at 24 h. All other Group 1 animals survived over 48 h. In sham mechanically ventilated controls not exposed to cotton smoke, there was no physiologic change over 48 h, with the PaO_2_/FiO_2_ ratio maintained at ≥ 500 and the static lung compliance was maintained throughout 48 h at about 1.5 mL/cmH_2_O/kg (not shown).

**Fig. 2:**
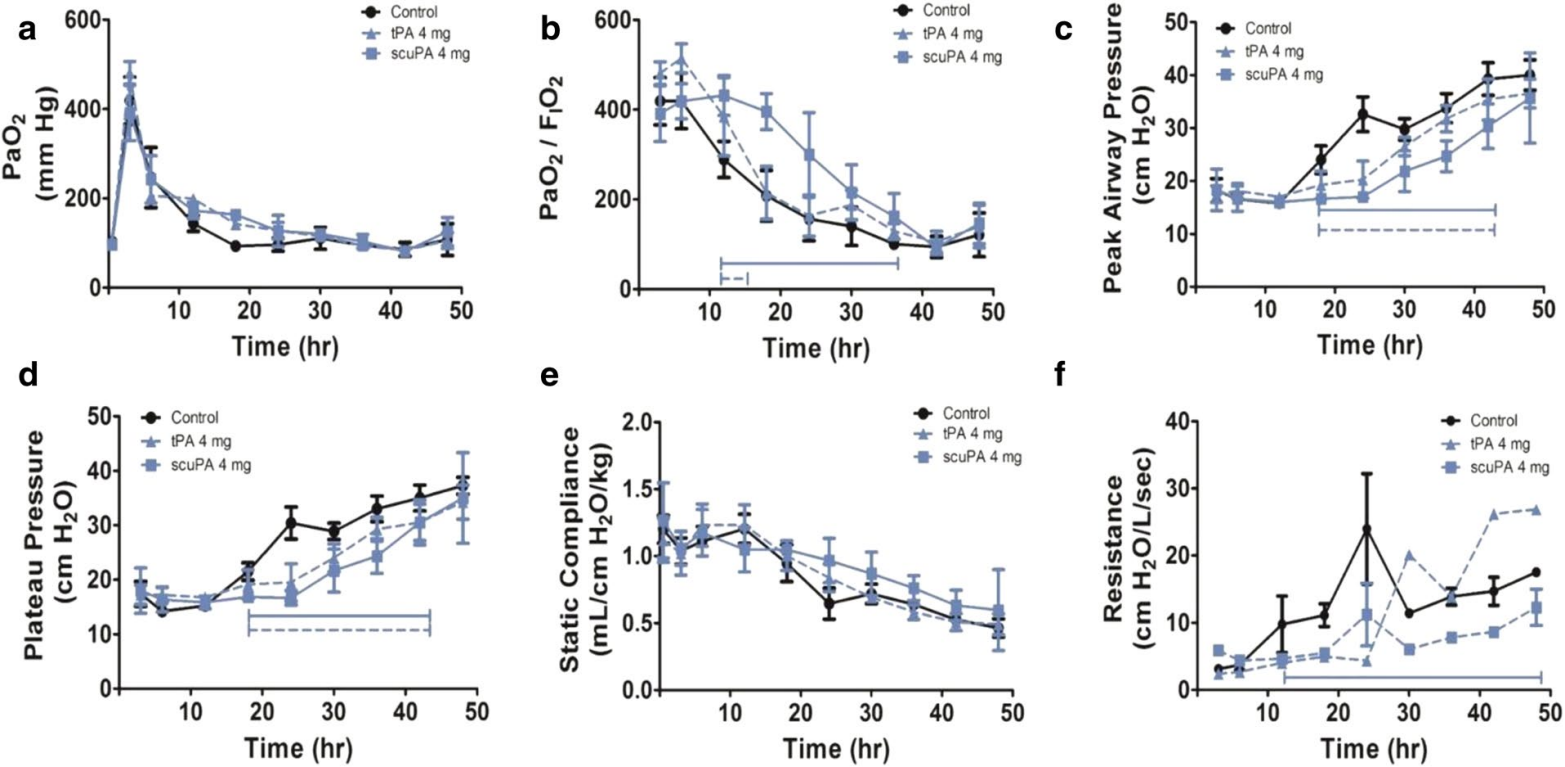
Group 1 pathophysiologic changes during the progression of ISIALI. Data from saline vehicle nebulized controls (n = 5, including one animal that died prematurely; before 48 h in the control vehicle group) or animals nebulized with tPA (n = 4) or scuPA (n = 3) in Group I at the 4 mg/unit dose are illustrated in **a**–**f** and plotted as line graphs with means and standard errors of the means. Nebulized plasminogen activators at the indicated doses were delivered according to the schedule illustrated in Fig. 1. **a** PaO_2_: arterial oxygen tension (mmHg): no significant difference between treatment groups over time. **b** PaO_2_/FiO_2_ ratios: statistically significant increase of scuPA (p = 0.02) and tPA (p < 0.001), compared to control. **c**, **d** Peak and plateau airway pressures (cmH_2_O): statistically significant decrement in tPA and scuPA treated groups compared to control (p < 0.001); scuPA treated was less than tPA (p = 0.03), independent of time. **e** Static lung compliance (mL/cmH_2_O/kg): no statistically significant difference between treatment groups as well as with control; **f** lung resistance (L/cmH_2_O/min): was significantly reduced in the scuPA 4 mg group (p = 0.045) but not in the tPA group. All depicted trends were statistically significant over time (p < 0.001). Statistical significance of treatment groups against controls over time is shown by solid (scuPA) or dashed (tPA) brackets

Following the administration of nebulized plasminogen activators (tPA and scuPA) at the 4 mg unit dose (Fig. 2a–f), there was a statistically significant increase in PaO_2_/FiO_2_ ratios over time to 48 h for animals treated with scuPA (p = 0.02) and tPA (p < 0.001), compared to controls. These were likewise significant increases than controls independent of time (p = 0.04 and < 0.001), respectively (Fig. 2b). Similar significant improvements occurred with decrements in the peak and plateau pressures (Fig. 2c, d, p < 0.001 in each case, independent of time) and the scuPA group demonstrated lower peak pressures than the tPA group (p = 0.03, independent of time, Fig. 2c). Lung compliance did not significantly differ between the sheep treated with nebulized plasminogen activators and saline vehicle-nebulized controls (Fig. 2e). Lung resistance was significantly reduced in the scuPA 4 mg group (p = 0.045, Fig. 2f) but not in the tPA group. At 48 h, the differences in PaO_2_/FiO_2_ ratios and all other physiologic parameters did not significantly differ between the treatment and control groups.

### Group 2: pathophysiologic responses to ISIALI and to nebulized plasminogen activator treatment

**Fig. 3:**
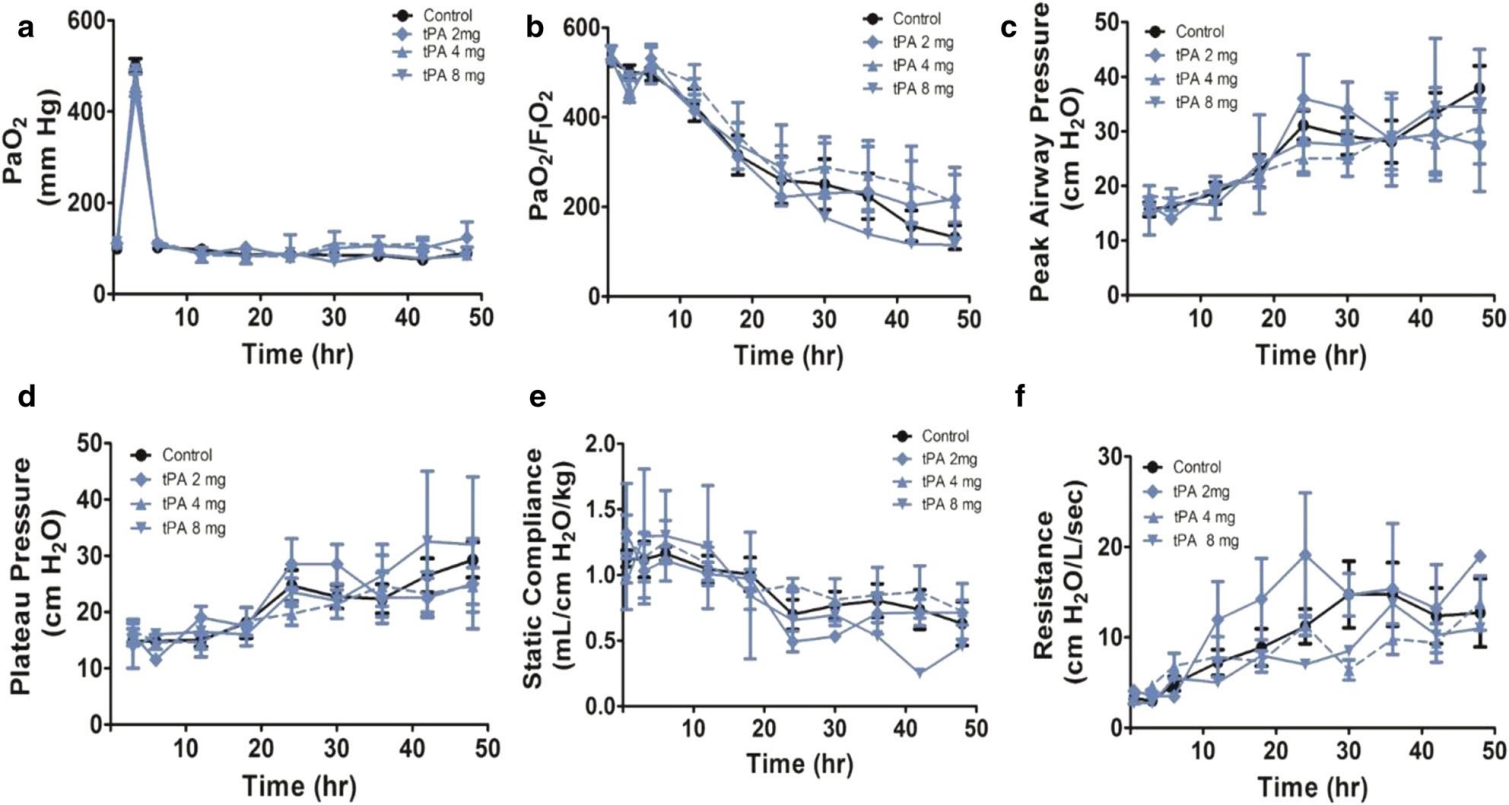
Pathophysiologic effects observed in Group 2 sheep treated with nebulized tPA. Data from saline vehicle nebulized controls (n = 9, including two animals that succumbed prematurely, prior to 48 h) or animals nebulized with tPA at the 2 mg dose according to the schedule illustrated in Fig. 1 (n = 2) or at the 4 mg/unit dose (n = 6, including one animal that succumbed prematurely) or at 8 mg (n = 2). **a** PaO_2_: arterial oxygen tension (mm Hg). **b** PaO_2_/FiO_2_ ratios. **c** Peak airway pressures (cmH_2_O). **d** Plateau airway pressures (cmH_2_O). **e** Static lung compliance (mL/cmH_2_O/kg). **f** Lung resistance (L/cmH_2_O/min). Although all the above trend over time were statistically significant (p < 0.001) there were no statistically significant intergroup differences

Given the potential for ventilator circuit losses (Additional file 1: Figure S1) and because pathophysiologic benefits of the nebulized PAs did not extend to 48 h in Group 1, we switched to the SCMV format and limited suction to augment airway delivery of the nebulized PAs (Group 2, n = 32 sheep). In our initial Group 2 experiments (Fig. 3a–f), we treated animals with 2 mg nebulized tPA (n = 2), which did not yield significant improvements in PaO_2_/FiO_2_ ratios, compliance or peak and plateau airway pressures versus vehicle-nebulized controls (n = 9, including two animals that were euthanized prior to 48 h). Interestingly, nebulized tPA at 4 mg (n = 6, including one animal that died before 48 h) was likewise ineffective in improving any of these same parameters versus controls (n = 9), so that the beneficial effects noted in Group 1 could not be corroborated in this larger comparison of sheep undergoing SCMV with reduced as needed, suctioning. Two animals were then treated with tPA 8 mg and each developed airway bleeding, including one animal that developed diffuse parenchymal bleeding involving all lobes and near occlusion of the airway with blood superimposed on uncleared airway casts. The second animal in this group developed airway hemorrhage that resulted in frank blood appearing in the ventilator tubing. This animal had grossly observed tracheal and large airway lesions (not shown). Both of these animals survived to 48 h, but there were no statistically significant improvements in oxygenation or other physiologic parameters.

**Fig. 4:**
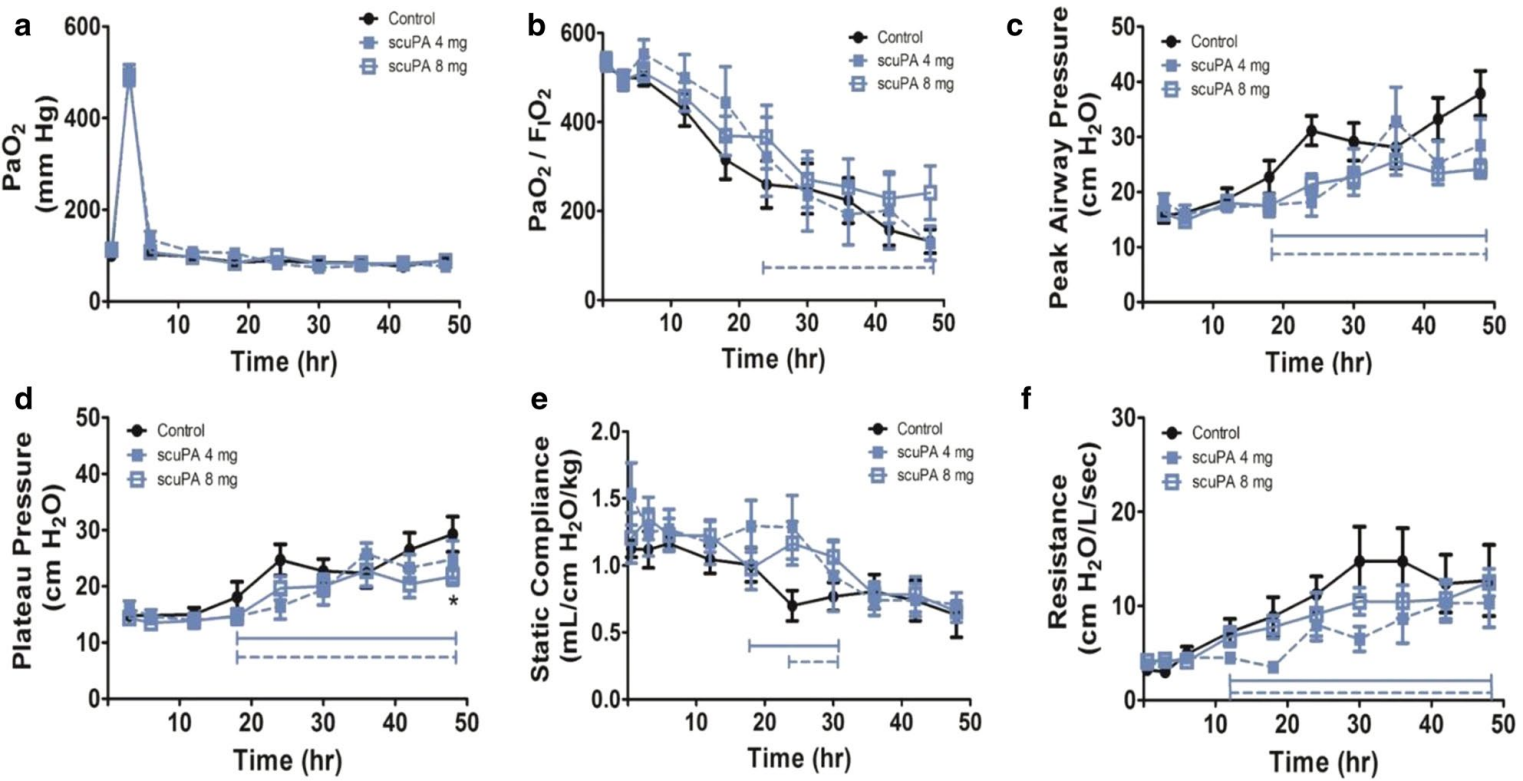
Pathophysiologic effects observed in Group 2 sheep treated with nebulized scuPA. Data from saline vehicle nebulized controls (n = 9) and from sheep treated with nebulized scuPA at the 4 mg (n = 5, including one animal that died prematurely) and 8 mg (n = 8, including one animal which died prior to 48 h) (Fig. 1). **a** PaO_2_: arterial oxygen tension (mmHg): no significant differences between dosing levels and control. **b** PaO_2_/FiO_2_ ratios: statistically significant increase for dosing at 8 mg compared to control (p = 0.02), independent of time; but no significant difference at 4 mg dosing. **c** Peak airway pressures (cmH_2_O) and **d** plateau airway pressures (cmH_2_O) were significantly reduced compared to controls over time (p < 0.001) at 8 mg and 4 mg scuPA. The asterisk in **d** shows a significant reduction of plateau pressures in the scuPA 8 mg group versus controls at the 48 h interval. **e** Static lung compliance (mL/cmH_2_O/kg) was greater compared with control overtime (p < 0.001), with scuPA 4 mg and scuPA 8 mg (p = 0.02 in each case) treated groups greater than control, independent of time. **f** Lung resistance (L/cmH_2_O/min) was significantly decrease compared to control overtime (p < 0.001), with scuPA 4 mg (p = 0.01) and scuPA 8 mg (p = 0.02) treated groups, independent of time. All depicted trends were statistically significant over time (p < 0.001). Statistical significance of treatment groups against controls is shown by solid (scuPA, 4 mg) or dashed (scuPA, 8 mg) brackets

The effects of scuPA nebulization in Group 2 sheep are shown in Fig. 4a–f. While there were no differences in arterial oxygenation (Fig. 4a), the PaO_2_/FiO_2_ ratios of the scuPA 8 mg (n = 8, including one animal that died prematurely) were increased significantly versus controls independent of time (n = 9, p = 0.02). The sheep treated with nebulized scuPA at 4 mg dosing did not differ from controls (Fig. 4b). Peak and plateau pressures were significantly reduced versus vehicle treated ISIALI controls by nebulized scuPA at 4 or 8 mg as a function of time, (p < 0.001) or independent of time (p < 0.001) in all cases except for the plateau pressure at scuPA 4 mg; p = 0.03, (Fig. 4c, d). Static lung compliance was increased in both the 4 and 8 mg scuPA groups versus vehicle nebulized controls independent of time during the 48 h course of progressive ISIALI (p = 0.02 in each case). Lung resistance was significantly reduced compared to control overtime (p < 0.001), with scuPA 4 mg (p = 0.01) and scuPA 8 mg (p = 0.02) treated groups, independent of time. By 48 h, only the plateau pressure of sheep nebulized with 8 mg scuPA remained significantly different from saline-treated controls and no overt bleeding into the airway or into the ventilator tubing was observed in sheep repeatedly treated at this nebulized unit dose.

### Change in the ventilation strategy to SCMV with limited suctioning generally increased BAL PA delivery

**Fig. 5:**
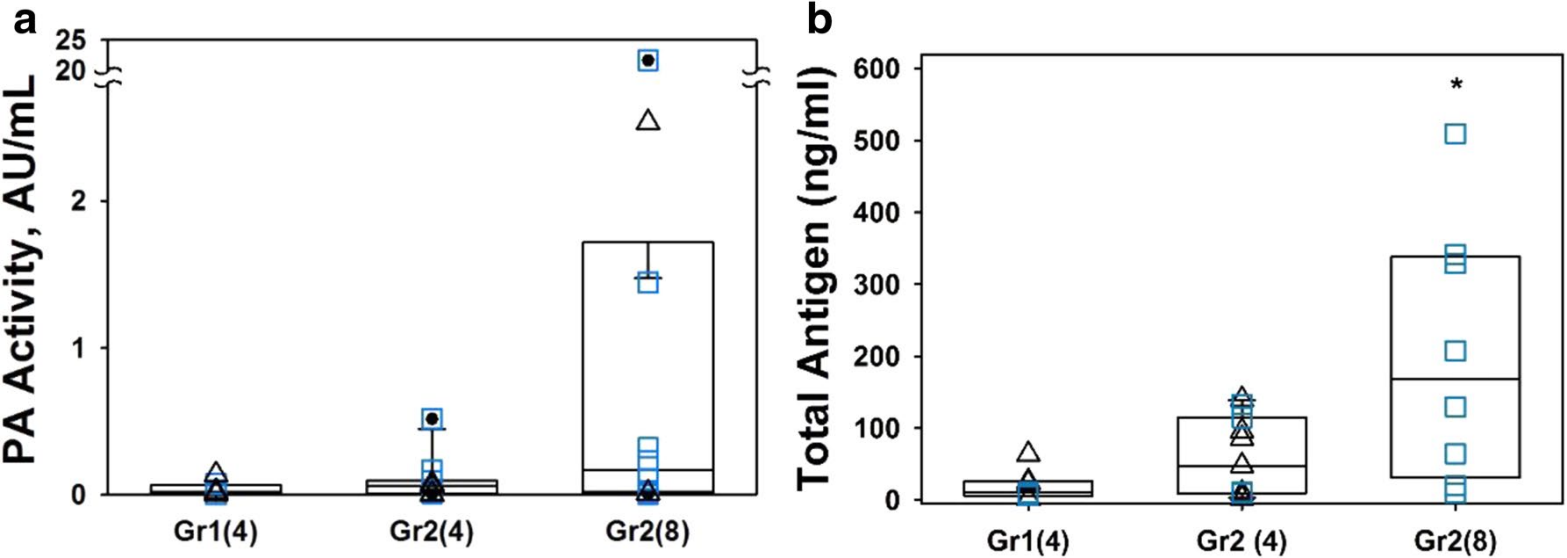
SCMV ventilation with limited suctioning and increased in a dose of plasminogen activator improved BAL PA delivery. **a**, **b** Changes in the ventilation strategy Group 2 (Gr2) vs Group 1 (Gr1) and an increase in the dose of the tPA (triangles) or scuPA (squares) from 4 to 8 mg resulted in an increase in BAL plasminogen activator (PA); human uPA or tPA activity (**a**) and antigen (**b**) after 48 h of treatment. Data are presented as box plots (showing interquartile ranges). Total antigen increases significantly (p < 0.001) overall as ventilation strategy changes from Gr1 at 4 mg through Gr2 at 4 mg to Gr2 at 8 mg. Pair-wise, the differences in median was profound only between Gr1 at 4 mg and Gr2 at 8 mg (p = 0.02). No statistically significant difference in the median of these parameters were noted between Gr1 and Gr2 at 4 mg. There was no statistically significant difference between levels of PA activity between the groups

Levels of PA activity in samples of BAL collected at 48 h after treatment with nebulized tPA and scuPA at the same 4 mg dose were generally higher in Group 2 using the SCMV/limited suction ventilation strategy (Fig. 5a). Further increments in BAL PA activity was observed with an increase in the dose of the plasminogen activator from 4 to 8 mg (Fig. 5a). Improved drug delivery was also observed at higher dosing levels based upon measurement of tPA and uPA antigen in BAL where total antigen increased significantly (p < 0.001) overall as ventilation strategy changes from Group1 at the dose 4 mg through Group 2 at 4 mg to Group 2 at 8 mg dose of plasminogen activators (Fig. 5b). No human uPA or tPA activity or antigen levels were detected in the BAL of control sheep treated with nebulized saline (n = 6), sham ventilated sheep without smoke exposure and no physiologic alterations over 48 h (n = 2) or naïve controls with no ISIALI nor mechanical ventilation (n = 3, data not shown).

### An increase in active plasminogen activators in BAL correlates with an increase in PaO_2_/FiO_2_ ratios

**Fig. 6:**
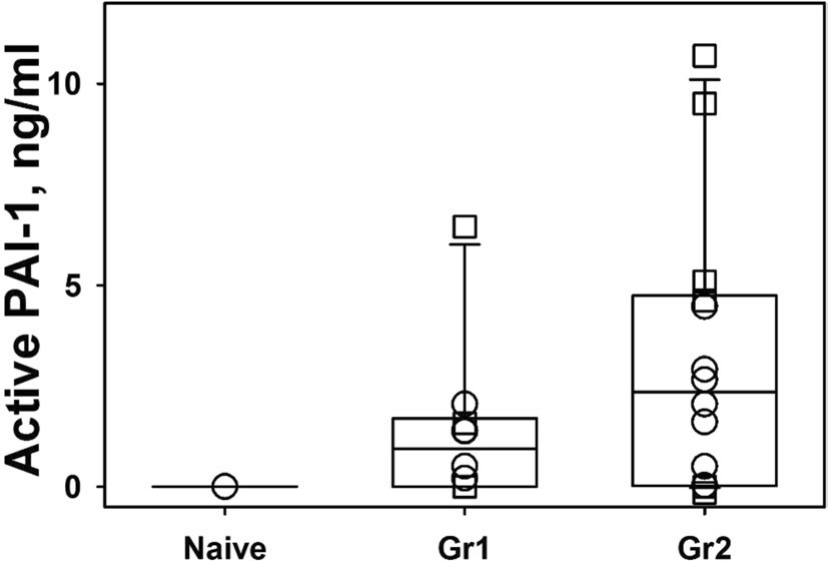
Levels of active PAI-1 in the BAL fluids of uninjured animals and animals with ISIALI. PAI-1 activity in BAL fluids of uninjured (naive; uninjured, not mechanically ventilated sheep, n = 3; sham; uninjured but mechanically ventilated sheep n = 2) animals and controls without treatment from Group 1 (GR1; n = 5) and Group 2 (GR2; n = 7) was measured using active rat PAI-1 ELISA (Molecular Innovations, Novi, MI; circles) and by titrating BAL fluid with uPA of a known concentration (squares) as previously described [15, 16]. The level of active PAI-1 in naïve (n = 3) and Group 1 sham (n = 2, not shown) was below level of detection using rat PAI-1 ELISA. Data are presented as box plots (showing median and interquartile ranges). There was no statistically significant difference between levels of active PAI-1 in the Group 1 versus 2 animals versus controls, although a trend towards increased BAL PAI-1 activity in sheep with ISIALI was observed

In additional subgroup analyses, there was a significant correlation (r = 0.83, p < 0.02) between improvement of the PaO_2_/FiO_2_ ratios and uPA-related activity in the BAL of scuPA 8 mg treated animals (n = 7). When the uPA activities of BAL 4 mg scuPA group (n = 4) and the 8 mg scuPA group (n = 7) from Group 2 were pooled, there was also a significant correlation; r = 0.71, between improvement of the PaO_2_/FiO_2_ and BAL uPA activity in the pooled group (n = 11; p < 0.001). In this group, the PaO_2_/FiO_2_ and BAL uPA antigen were also significantly correlated (r = 0.88, p < 0.001). Interestingly, the correlation (r = 0.98) between PaO_2_/FiO_2_ ratios and total BAL tPA activity of the tPA 4 mg nebulized sheep (n = 4) was likewise statistically significant (p < 0.001). However, there was no significant correlation; r = 0.23, p = 0.70, between improvement of the PaO_2_/FIO_2_ ratio and total tPA antigen in the tPA 4 mg treated animals. Active PAI-1 was readily detectable in the BAL in control saline-treated animals with ISIALI (Fig. 6).

**Fig. 7:**
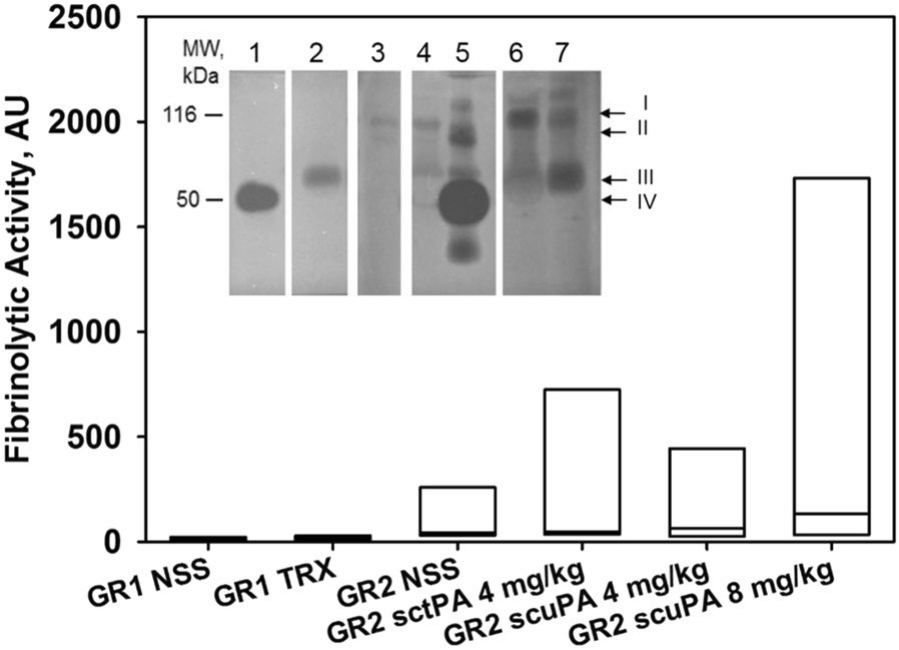
Assessments of fibrinolytic activity in BAL samples. Generation of fibrinolytic activity by BAL fluids in the presence of human plasminogen. Box plots: relative fibrinolytic activity (FA), produced by BAL fluids (Gr1: 5 saline-controls, GR1 TRX: 11 scuPA and tPA treated animals; Gr2: 6 saline controls, 5 and 7 animals treated with 4 and 8 mg of nebulized scuPA respectively, and 5 animals treated with 4 mg nebulized tPA) in the presence of 100 nM human Glu-plasminogen. Data are presented as box plots (showing interquartile ranges). No statistically significant differences were noted in the median value of FA between the groups. Inset: enzymographic analyses of BAL fluids from animals with ISIALI. BALs were collected at 48 h after initiation of the treatment at the time of euthanasia. Arrows to the right of the panels indicate positions of: tPA/PAI-1 inhibitory complexes (110 kDa) (I), uPA/PAI-1 inhibitory complexes (100 kDa) (II), tPA (63 kDa) (III), uPA (50 kDa) (IV). To illustrate the range of activity in the treatment groups, samples the range of ones with low levels of PA and relatively higher PA levels from sheep included in Group 2. Lanes: recombinant human uPA (0.5 ng) and tPA (0.1 ng) standards are shown in lanes 1 and 2, respectively. 3: saline treated animal, 4 and 5—8 mg scuPA-treated animals, 6 and 7—4 mg tPA-treated animals. The data are representative of the findings in all similarly treated Group 2 animals

**Fig. 8:**
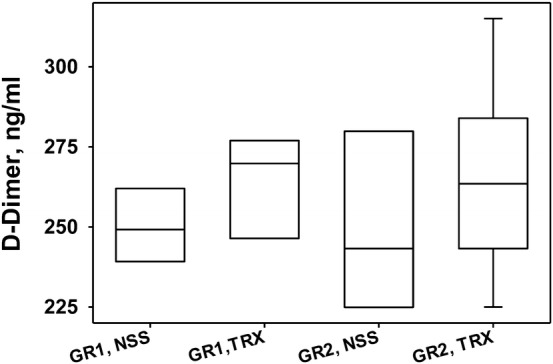
The effect of fibrinolytic therapy on concentrations of d-dimers in BAL of Groups 1 and 2. The concentration of d-dimer in BAL of ISIALI sheep after fibrinolytic therapy was measured using a sheep d-dimer (D2D) ELISA Kit (MyBioSource, CA) as described in “Methods”. Sheep with induced smoke ALI was treated with nebulized scuPA (4 mg) or tPA (4 mg) (Gr1, TRX) and scuPA (4 mg or 8 mg) or tPA (4 mg) (GR2 TRX) or vehicle control (Gr1, NSS and Gr2, NSS). BAL (Gr1: 5 saline-controls, GR1 TRX: 11 scuPA and tPA treated animals; Gr2: 6 saline controls, 5 and 7 animals treated with 4 and 8 mg of nebulized scuPA respectively, and 5 animals treated with 4 mg nebulized tPA) was collected at 48 h after the initiation of ISIALI as described in Fig. 1 and then analyzed [23]. Data are presented as box plots (showing interquartile ranges). No statistical significant difference was observed in the median values of d-dimer between treatment groups

We next sought to assess fibrinolytic activity readouts in the BAL and initially used fibrin enzymography to detect zones of fibrinolysis associated with the nebulized PAs; tPA or scuPA. In the treatment groups in Group 2, a trend towards increased BAL fibrinolytic activity was observed in the tPA or scuPA groups at 4 h after the last administered dose (Fig. 7). Fibrinolytic activity was not detected in sham or naïve controls (n = 2 and 3 respectively, data not shown). By fibrin enzymography, zones of fibrinolysis associated with tPA or uPA were identified in all of the sheep treated with either nebulized tPA or scuPA 4 mg in Groups 1 and 2 and surviving to 48 h (Fig. 7, inset). No such bands were identified in the control saline-treated animals (Fig. 7, inset, lane 3). These data indicate that active plasminogen activator was identified in the BAL of sheep in which ISIALI was induced and that were then treated with nebulized tPA or scuPA at this dose. We next performed an independent analysis of fibrinolytic activity in BAL and detected low levels by fluorogenic analyses in some of the animals treated with either tPA or uPA in Group 2 (data not shown). The results are likely in part attributable to the lesser sensitivity of this assay versus that of fibrin enzymography in which the detector gels lyse over the course of 24 h or more. Plasminogen supplementation was next used to augment the fibrinolytic signal, since BAL represents a dilute form of extravascular lung lining fluid in which low plasminogen levels could limit detection of fibrinolytic activity. When supplemented by plasminogen, the differences in the fibrinolytic activities of the BAL samples of the tPA or scuPA-treated sheep became more apparent (Fig. 7, bar plots). Although there were no significant differences in fibrinolytic activity of the BAL from the nebulized tPA or scuPA groups, greater signal was detected in BAL of the animals from Group 2. There was a trend of increased levels of fibrinolytic activity in the scuPA or tPA-treated animals versus saline-treated controls in Group 2. d-Dimer levels in the BAL of sheep from these treatment groups exhibited the same trends (Fig. 8).

### Group 2 lung tissue injury outcomes

All cotton-smoke exposed animals had characteristic changes associated with ISIALI, with histologic changes consistent with our prior reported findings in the model, with scoring of the extent of injury done as we previously described [5]. Representative histologic images from control and treated animals in Group 2 are provided in the Additional file 1: Figure S2. There were no significant changes between the saline vehicle control-treated sheep versus those treated with nebulized tPA or scuPA with regard to alveolar edema, alveolar neutrophil, overall septal cell infiltration, atelectasis or major bronchial inflammation or bronchial or bronchiolar obstruction scores. In the tPA 8 mg group, one animal with extensive airway and parenchymal lung hemorrhage had focal necrotic areas in the trachea with a suggestion of bacterial invasion that can occur in the model. Similar lesions were identified in the major airways of other animals in Group 2 that did not experience hemorrhage. The second animal that had blood in the ventilator lines had grossly observed areas of focal large airway hemorrhage at post-mortem examination. Residual large airway casts of varying sizes were observed in most sheep with ISIALI at the time of gross post-mortem examination (not shown).

### Plasma levels of uPA, tPA, d-dimer and αM/uPA complexes

**Fig. 9:**
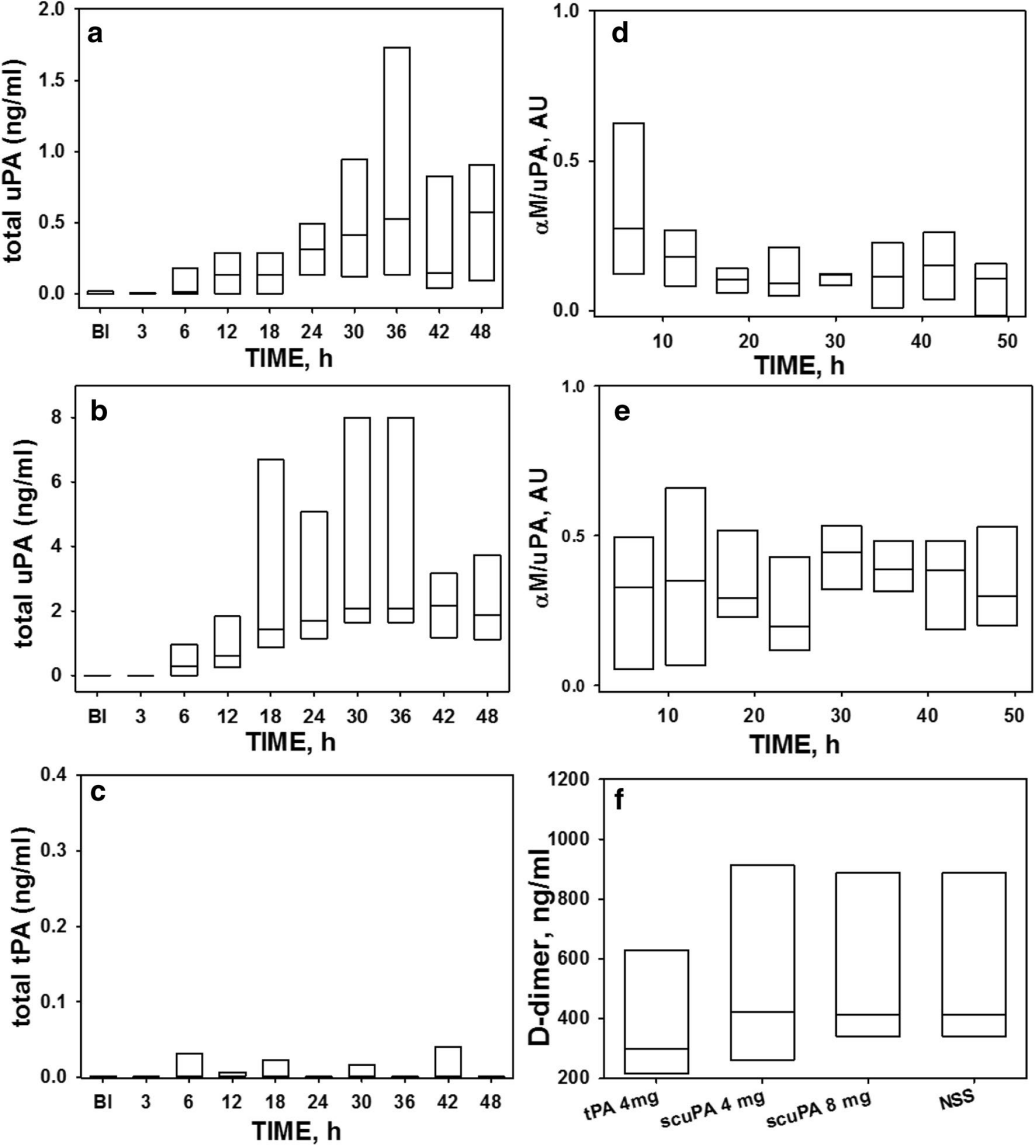
Effect of nebulized scuPA and tPA treatments on detection of the plasminogen activators, αM/uPA complexes and d-dimers in plasma. The data shown were obtained from all animals in Group 2 and illustrated in a box plots (showing median with interquartile range). Temporal changes in the levels of uPA antigen (**a**, **b**) and “molecular cage” type αM/uPA complexes (**d**, **e**) in plasma of animals treated with 4 and 8 mg scuPA are illustrated, respectively. Levels of uPA antigen (**a**, **b**) in plasma of animals treated with 4 and 8 mg scuPA increase significantly over time (p < 0.001). Difference were more profound with higher levels for animals treated with scuPA at 8 mg compared with those treated with tPA (**c**) or scuPA (**a**) at 4 mg at each time point where samples were collected, while levels for the scuPA at 4 mg were not significantly different from those for tPA (**c**). There was no statistical significant difference between plasma levels of d-dimers (**f**), indicating comparable systemic fibrinolysis in all treatment groups. **c** Illustrates relatively low total tPA antigen levels including all animals from the Group2 4 mg (n = 5) subgroups. Data are presented as box plots (showing interquartile ranges). The concentrations of antigens were determined by ELISA assays designed to detect the human antigens (Molecular Innovations, Inc., MI). Levels of αM/uPA complexes were assessed by uPA amidolytic activity in the plasma in the presence of 100 nM human recombinant PAI-1, as described [24]. The concentration of d-dimer in plasma of ISIALI sheep (**f**) after vehicle, tPA or scuPA nebulized treatment was measured using sheep d-dimer (D2D) ELISA Kit (MyBioSource, CA) as described in “Methods”

We lastly measured uPA and tPA antigens in the plasma of the sheep treated with nebulized Group 2 scuPA, tPA and saline controls (Fig. 9a–f). In saline-nebulized controls with ISIALI, no human tPA or scuPA was detectable in plasma, as anticipated (data shown). Levels of uPA antigen (Fig. 9a, b) in plasma of animals treated with 4 and 8 mg scuPA, respectively, increase significantly over time (p < 0.001). Difference were more profound for animals treated with scuPA at 8 mg compared with those treated with tPA or scuPA at 4 mg at each time point where samples were collected, while levels for the scuPA at 4 mg were not significantly different from those tPA at 4 mg (Fig. 9a–c). Detectable levels of αM/uPA complexes were found in the scuPA-nebulized animals (Fig. 9d, e) with the greatest levels observed in animals that received the highest; 8 mg dose of scuPA. These complexes were not found in the plasma of sheep treated with nebulized tPA (data not shown). Interestingly, a relatively high level of plasma tPA; 0.24 ng/mL at 48 h, was found in the tPA 8 mg animal that experienced major airway hemorrhage proximate to this determination. Plasma levels of d-dimer were not significantly different between controls and either the scuPA or tPA-nebulized sheep (Fig. 9f). Of particular note, d-dimers were not increased in conjunction with bioactive αM/uPA complex formation in plasma of sheep treated with nebulized scuPA.

## Discussion

We found that airway delivery of either tPA or scuPA was well-tolerated at doses of 2 or 4 mg mg/unit dose delivered every 4 h during the development of ISIALI in mechanically ventilated sheep. Doses of scuPA were likewise well-tolerated at 8 mg/nebulized treatment that was delivered every 4 h. There was no significant difference in the mortality of animals that received nebulized vehicle, saline, or those that received nebulized either scuPA or tPA at these doses. In Group 1, one control animal had to be euthanized and two sheep receiving 2 mg tPA dosing likewise did not survive to 48 h, attesting to the severity of the injury model we used, which was characterized by extensive fibrinous cast formation in the upper airways. Injury severity was clearly increased in the current study, by virtue of a generally earlier decrement in the PaO_2_/FiO_2_ ratios in the current study (Figs. 2b, 3b) and no improvement in the airway obstruction scores after treatment with nebulized plasminogen activators every 4 h versus that done in 2004 [5]. The disparate findings between the present and 2004 studies in which efficacy of this regimen was described could be due to subtle differences in the severity of injury, intensity of airway toilet, the smoke generated by different batches of cotton bark or variable responses of individual outbred sheep to the smoke used to induce ISIALI, which could likewise vary between herds. The disparity appeared to be unrelated to loss of activity of nebulized material. We posit that the different outcomes more likely relate to the severity of ISIALI in the model as currently used.

Levels of tPA or scuPA antigen were often undetectable in the BAL obtained from the Group 1 animals, suggesting that losses either via nebulization or in the ventilator circuit could have contributed to the transient improvements we saw in the PaO_2_/FiO_2_ ratios and other physiologic outcomes (Fig. 2). The low plasminogen activators yields in BAL appeared to be unrelated to loss of activity by nebulization. Formulation in saline was not likely responsible, as tPA activity (and that of nebulized scuPA) was preserved. Nebulization of tPA and scuPA in the Aerogen nebulizer yields approached 90% of the nebulizer charge, which suggested that drug losses within the ventilator tubing circuit or via suctioning were more likely to have been responsible Using a simulation of the APVcmv ventilator circuit, we found that such losses occurred (Additional file 1: Figure S1).

Based on these considerations, we repeated the dose escalations of nebulized tPA and scuPA in a second, larger group of sheep with ISIALI that were ventilated using CMV mode with limited suctioning (Group 2). The generally greater BAL human PA activity and antigens suggests improved delivery in these animals. In Group 2, there were no significant changes in the pathophysiologic outcomes of ISIALI at 48 h between control saline vehicle-treated animals (n = 9) and those nebulized with tPA at a unit dose of 2, 4 or 8 mg/nebulized treatment with respect to the PaO_2_/FiO_2_ ratios, airway pressures, lung compliance or resistance (Fig. 3). Conversely, in Group 2 there were small but significant benefits of intervention with either 4 or 8 mg of scuPA in that the PaO_2_/FiO_2_ ratios were increased over the course of injury versus controls (p < 0.01), while the peak and plateau pressures of animals treated with both the 4 and 8 mg doses of scuPA were significantly reduced versus controls (p < 0.01 in each case). Lung compliance likewise was increased in both the 4 and 8 mg scuPA-treated animals versus controls (p < 0.01). These small effects were almost completely obviated at 48 h, indicating that the physiologic improvements of nebulized scuPA versus tPA were transient and could be overcome by progressive, severe ISIALI by 48 h. The lack of morphometric changes in the severity of ISIALI at 48 h in animals treated with nebulized plasminogen activators versus vehicle controls aligns with the lack of durable pathophysiologic improvements and may have been due to insufficient activation of sheep plasminogen by the human PAs within alveolar lining fluids or airway casts. A limitation of our study is that overall airway cast burden could not reliably be determined, as large, obstructing casts were suctioned to ensure animal survival.

Interestingly, dose escalation of tPA to the 8 mg/nebulized dose resulted in massive parenchymal lung hemorrhage in the first animal we treated and overt bleeding into the tubing of the ventilator circuit in the second animal treated at the same dose. Given these sequential, sentinel bleeding outcomes and the imperative to conserve animals and cost, allocation of additional animals to the tPA 8 mg group was not felt to be justified. These results are consistent with the pulmonary bleeding demonstrated in mice exposed to dose escalations of tPA by intratracheal nebulized airway delivery [7] and suggest that tPA was active after airway delivery. It is possible that airway bleeding may have been promoted by the relatively greater fibrin specificity of tPA and localization to friable airway injury. The findings also leave open the possibility that dosing of nebulized tPA at > 4 mg but < 8 mg may have been more effective.

No systemic bleeding was observed in any of the animals treated with nebulized tPA or scuPA, suggesting that bleeding risk associated with airway administration is mainly localized to the airways or lung parenchyma. Detectable uPA antigen was often detected in the plasma of sheep treated with nebulized scuPA (Fig. 9a, b). The detection of increased levels of bioactive αM-uPA complexes in these animals suggests that inhaled scuPA undergoes processing that promotes their formation. These complexes likely derive from egress from the injured lung. d-Dimers were not increased in the BAL or plasma of these animals, possibly due to the severity of lung inflammation or inefficient fibrinolysis.

While the biochemical BAL data shows that exposure to airway plasminogen activators was generally improved in Group 2, limitation of physiologic improvement to the highest dose of nebulized scuPA suggests the possibility that ongoing airway coagulation with cast formation could have blunted the responses. Fibrinous cast formation was observed grossly in the airways at euthanization or at completion of the 48 h course of ISIALI in each animal and as we previously reported [5], and the casts were usually not fully cleared by administration of PAs. These findings in this study suggest that the administration of plasminogen activators in this study was insufficient to lyse airway clots to the extent that physiologic outcomes were favorably impacted at 48 h after induction of ISIALI. It is also possible that species cross reactivity could have limited activation of sheep plasminogen by the nebulized PAs, but in our previous study [5], we found that tPA lysed airway clots effectively and improved outcomes with less severe ISIALI. This work offers proof of concept that a human PA; tPA, can activate endogenous sheep plasminogen and resolve airway clots and we infer that scuPA, in part through conversion to two chain uPA, can exert similar effects since transient improvements were observed using nebulized scuPA. It is lastly conceivable that nebulized PAs favored distal mobilization of cast fragments, generating new ventilation/perfusion imbalances, thereby obviating potential benefits. Exploration of this possibility is beyond the scope of this study and would require future dedicated studies.

PAI-1 was previously shown to be overexpressed in the lungs in ISIALI [17] and was confirmed in the current model. These data are consistent with prior observations demonstrating robust expression of PAI-1 in lavage fluids from animals with ALI and in BAL collected from patients with a variety of forms of ARDS [17–20]. Our data extends these observations by showing that active PAI-1 occurs in BAL in this ISIALI model, which is to our knowledge the first such observation in the field.

It should be noted that the ability to disperse airway casts clearly contributes to the salutary effects of airway delivery of tPA in sulfur mustard-induced acute lung injury, which is likewise characterized by fibrinous cast formation [6]. In this study, we found that dose escalations of nebulized scuPA conferred transient benefits that exceeded those of tPA but were likewise obviated by the progression of ISIALI. scuPA was previously nebulized by others, with partial reversal of suppressed fibrinolysis in acutely injured lungs and no induction of systemic fibrinolysis [21, 22]. Overall, we found that nebulized tPA or scuPA did not confer durable benefits in the ISIALI model in sheep under the conditions used in this study.

## Additional file

**Additional file 1.** Supplemental data.
