# Supplementary material for: Lack of durable protection against cotton smoke-induced acute lung injury in sheep by nebulized single chain urokinase plasminogen activator or tissue plasminogen activator
Source: Clin Transl Med. 2018 Jun 18;7:17. doi: 10.1186/s40169-018-0196-3 (PMC6006005; doi:10.1186/s40169-018-0196-3)
Supplement: Supplementary file 1 — Additional file 1. Supplemental data. [file 40169_2018_196_MOESM1_ESM.docx]

**Additional file 1**

Lack of Durable Protection Against Cotton Smoke-Induced Acute Lung Injury in Sheep by Nebulized Single Chain Urokinase Plasminogen Activator or Tissue Plasminogen Activator by Fukuda S. et. al.

**Figure S1. Simulation Circuit Components.**

**
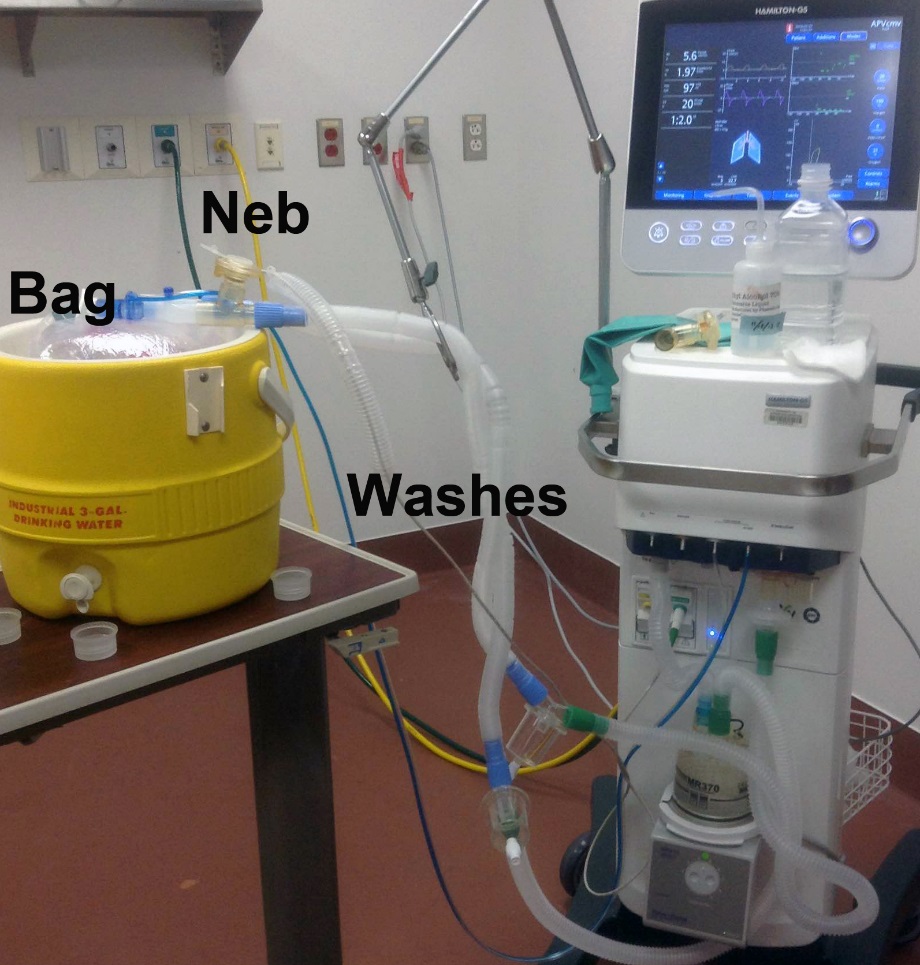
**

**Legend.** The experimental setup for nebulization of Activase/alteplase; tPA or scuPA into a one gallon plastic bag intended to simulate delivery to the lungs is illustrated in the photograph. Samples of 4 mg of the plasminogen activator (tPA or scuPA; n=2/condition) in 8 mL of PBS were nebulized (Neb) and delivered via ventilator into a plastic bag (Bag) on ice. The tidal volume was 100 ml, oxygen level was either 21% (with or without humidifier) or 100% (with humidifier). Protein and enzymatic activity levels were measured in samples collected from the bag, and in 200 mL of DPBS wash solution of ventilator tubing (Washes), and compared to 100% of initial activity and protein placed into the nebulizer. While nebulization and delivery into 1 gallon bag did not affect the specific enzymatic activity of either plasminogen activator, it was decreased in the samples from DPBS after washing the tubes.

**1.** **Ventilator simulation circuit details.** In this model, a 1 gallon insulated plastic bag maintained on ice was used to capture the condensate of nebulized solutions of sctPA and scuPA that were generated by the Aerogen nebulizer; the Aeroneb® Pro (Aerogen, Mountain View, CA). Breaths were delivered by the same Hamilton-G5 ventilator in the APVcmv mode as used in the Group 1 animals. The ventilator was set at a rate of 20 breaths/minute and a tidal volume of 100 cc, which accounted for full expansion of the bag. Samples of 4 mg of sctPA or scuPA in 8 ml of PBS were nebulized in these experiments. Three combinations of conditions (21% oxygen with or without humidifier in room air and 100% of oxygen with humidification generated by a MR410 humidifier (Fisher and Paykel Healthcare, Inc., Irving, CA,) were carried out in duplicate for each enzyme (total n=12 independent experiments). An additional experiment was performed where 10 ml of phosphate-buffered saline, PBS, was added to the bag prior to nebulization (total n=3 independent experiments) in an attempt to increase the yield of the drug captured in the bag. To screen for protein that was not captured in the bag, outflow tubing was washed with 200 ml of sterile PBS and collected for analysis. Aliquots of samples from the nebulizer, bag and washes were analyzed for amidolytic activity and protein concentration in order to determine efficacy of delivery and effects of oxygen and humidification on the nebulized plasminogen activators. The total amounts of nebulized protein (mg), amidolytic and specific activities of each enzyme (Arbitrary Units of Activity; AU) were then calculated for all samples collected prior to and after nebulization. The nebulizer was confirmed to be empty at the conclusion of each experiment.

**Assessments of ventilator circuit plasminogen activator losses in the APVcmv ventilator simulation model.**

We first sought to confirm that nebulization of the plasminogen activators did not the affect the specific activity of the enzymes. Nebulization and mechanical ventilation with or without an in-line humidifier and 21 or 100% oxygen did not affect the specific amidolytic activity of sctPA or scuPA recovered in the 1 gallon bag. Recovered specific activities versus that of material loaded into the nebulizer of the plasminogen activators were=103±32% (mean±SE). No self-activation of scuPA was detected in the samples captured into the bag and humidification decreased the concentration of the nebulized plasminogen activator in the bag approximately two times without affecting the specific activity (data not shown). While the specific activity of each plasminogen activator was preserved during nebulization, only a small fraction; 13.0±12.2% of the initial amount of sctPA or scuPA was recovered from the bag, confirming loss of plasminogen activators within the circuit. PBS added to the bag did not affect yield. Significant amounts of plasminogen activators; either tPA or scuPA (60.4±18.2%) were found in washes harvested from the expiratory tubing. Since the balance of the nebulized agents were not detected in either the repository bag or in lavage of the expiratory ventilator circuit tubing, additional losses may have occurred elsewhere within the circuit, including losses adherent to tubing or external leakage through seals. These results excluded inactivation of the plasminogen activators during nebulization and suggested that losses could occur in the ventilator circuit.

**Figure S2. Histologic analyses of lung parenchymal and airway injury observed in ISIALI.**


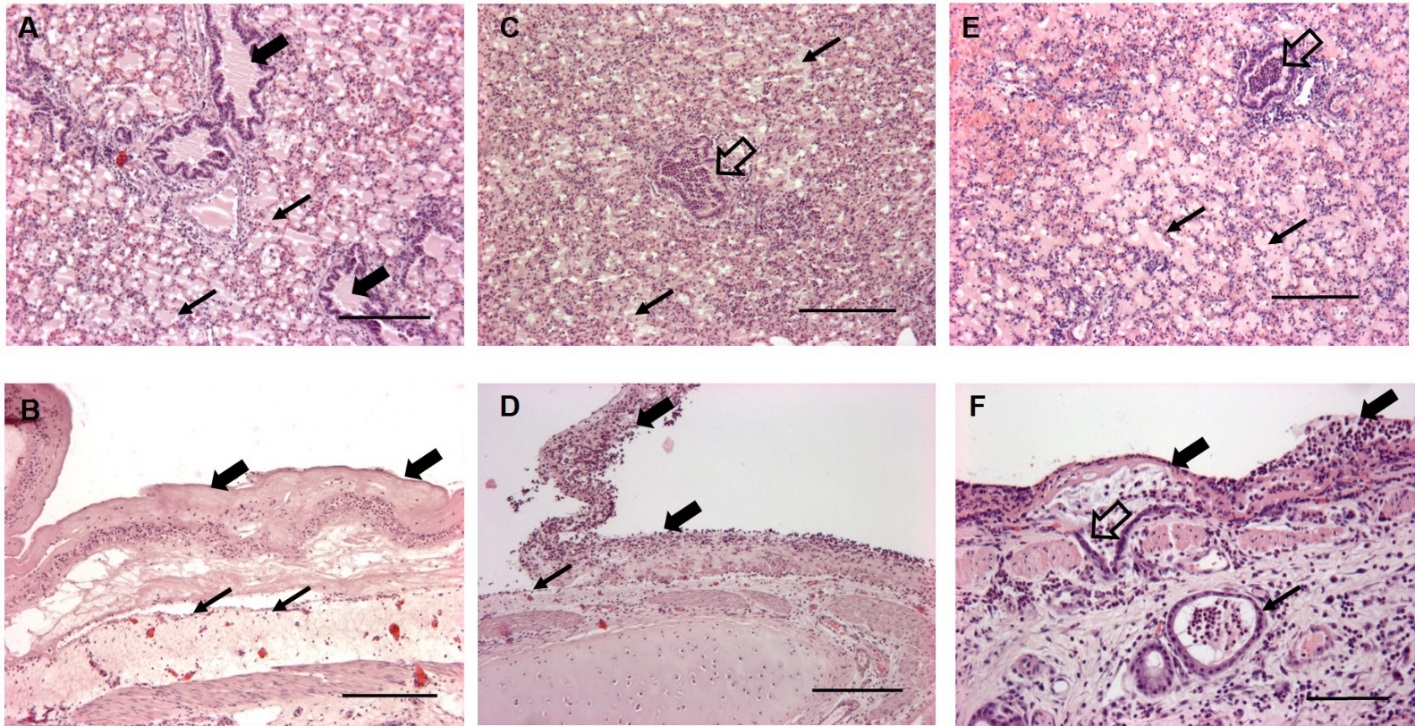


**Legend.** Representative lung tissue sections of Group 2 sheep treated with saline vehicle control (**A and B**), 4 mg of tPA (**C and D**) or 4 mg scuPA (**E and F**) were stained with hematoxylin and eosin. **Top**: Alveolar edema and neutrophilia denoted with fine arrows. Focal areas of the lung show bronchioles filled with exudative material indicated with large filled arrows. The empty arrow in Panels C denotes an obstructed bronchiole filled with inflammatory cells and exudate. Bar = 200 micron. **Bottom**: **B and D.** Broad arrows indicate eosinophilic exudate containing inflammatory cells at the surface of denuded bronchial walls, associated with epithelial cell loss. Sloughed necrotic epithelium is denoted by fine arrows. Gland neutrophilia is seen in the mucosal area (fine arrow, **F**). The V like structure in Panel F represents the interface of the ciliated duct mucosal epithelium with the lumen (open arrow, **F**). Bar = 200 micron (**B and D**), 100 micron (**F**). Comparable patterns of lung parenchymal and airway injury were observed in all animals in Group 2.
